# Pharmacokinetics of post-transplant cyclophosphamide and its associations with clinical outcomes in pediatric haploidentical hematopoietic stem cell transplantation

**DOI:** 10.1186/s40364-025-00749-3

**Published:** 2025-03-24

**Authors:** Kyung Taek Hong, Sungyeun Bae, Yoon Sunwoo, Juyeon Lee, Hyun Jin Park, Bo Kyung Kim, Jung Yoon Choi, Joo-Youn Cho, Kyung-Sang Yu, Jaeseong Oh, Hyoung Jin Kang

**Affiliations:** 1 Department of Pediatrics, Seoul National University College of Medicine, Seoul National University Children’s Hospital, Seoul, Republic of Korea; 2 Seoul National University Cancer Research Institute, Seoul, Republic of Korea; 3 Department of Clinical Pharmacology and Therapeutics, Seoul National University College of Medicine and Hospital, Seoul, Republic of Korea; 4 Department of Biomedical Sciences, Seoul National University College of Medicine, Seoul, Republic of Korea; 5 Department of Pharmacology, Jeju National University College of Medicine, Jeju, 63241 Republic of Korea; 6 Wide River Institute of Immunology, Hongcheon, Republic of Korea

**Keywords:** Haploidentical stem cell transplants, Cyclophosphamide, Pediatrics, Pharmacokinetics, Clinical outcomes

## Abstract

**Background:**

Post-transplantation cyclophosphamide (PTCy) has paved the way for the increased use of alternative donors, including haploidentical familial donors, with acceptable engraftment and graft-versus-host disease (GVHD) rates. However, pharmacokinetic studies of PTCy in the pediatric population following myeloablative conditioning regimens are scarce.

**Methods:**

We conducted a prospective and comprehensive pharmacokinetic analysis of pre- and post-transplantation cyclophosphamide levels in pediatric patients undergoing haploidentical hematopoietic stem cell transplantation (HSCT) using a myeloablative busulfan-based conditioning regimen. A total of 14 samples were collected from each patient. Plasma concentrations of cyclophosphamide and carboxycyclophosphamide were analyzed, and clinical outcomes were recorded. The simulated pharmacokinetic profiles of cyclophosphamide and its metabolites were compared among different age groups using real-world data.

**Results:**

A total of 15 pediatric patients (median age at HSCT 9.6 years, range 1.6–16.8) were enrolled. Thirteen patients had malignant disease. All patients achieved successful neutrophil engraftment, and the cumulative incidences of grade 2–4 acute GVHD and moderate-to-severe chronic GVHD were 13.3% and 14.7%, respectively. The patterns of cyclophosphamide pharmacokinetic parameters were similar between the pre- and post-HSCT doses. The metabolic ratio increased with subsequent doses of PTCy. Patients with severe veno-occlusive disease showed a higher cumulative area under the curve (AUC) of carboxycyclophosphamide (62.6 vs. 40.2 mg x h/L, *P* = 0.025), while patients with > grade 3 hemorrhagic cystitis had a higher cumulative AUC of cyclophosphamide (1256.2 vs. 778.2 mg x h/L, *P* = 0.009). In contrast, there were no notable differences in the pharmacokinetic parameters of cyclophosphamide and carboxycyclophosphamide between the groups with and without acute and chronic GVHD. The AUC of cyclophosphamide and its metabolite were similar in children weighing ≥ 30 kg and the virtual adult population.

**Conclusions:**

Our study provides insights into the pharmacokinetic profile of cyclophosphamide and its metabolite, carboxycyclophosphamide, in pediatric patients undergoing haploidentical HSCT with PTCy. The intricate interplay between pharmacokinetic parameters and post-HSCT complications suggests the need for tailored adjustments in PTCy dosage, particularly in pediatric patients subjected to myeloablative conditioning regimens.

**Supplementary Information:**

The online version contains supplementary material available at 10.1186/s40364-025-00749-3.

## Introduction

Allogeneic hematopoietic stem cell transplantation (HSCT) is a curative treatment for high-risk hematological malignancies, bone marrow failure, and immunodeficiencies. When matched donors are unavailable, haploidentical donors—such as haploidentical siblings or parents—serve as vital alternatives. To address challenges like graft-versus-host disease (GVHD) and engraftment failure, various strategies have been employed in haploidentical HSCT [1]. Among these, T cell-replete haploidentical HSCT using post-transplant cyclophosphamide (PTCy) has emerged as a promising approach, simplifying the application of haploidentical donors in allogeneic HSCT [2]. Initially developed in conjunction with a non-myeloablative conditioning regimen and bone marrow as the stem cell source for adult patients, this approach has shown positive outcomes in diverse settings [3], even in the context of HSCT with matched donors [4]. Studies in pediatric patients have also demonstrated its effectiveness [5–7], particularly when combined with myeloablative conditioning regimens [8–11]. Notably, our center has previously reported promising results from PTCy-based haploidentical HSCT using a targeted busulfan-based myeloablative conditioning regimen [7, 12]. As a result, the use of PTCy is expected to increase in various HSCT.

The pharmacokinetics (PK) of Cy and its metabolites are related to clinical outcomes in HSCT patients. A recent study found that carboxy-ethyl phosphoramide mustard (CEPM) PKs in adult patients undergoing haploidentical HSCT were associated with severe chronic GVHD and GVHD-/relapse-free survival [13]. In addition, Salinger et al. suggested that exposure to hydroxycyclophosphamide (HCY) and CEPM was associated with efficacy and toxicity being proposed real-time dose adjustment of Cy using a Bayesian PK approach [14]. Though there have been PK studies on Cy in pediatric patients [15], there is currently no research on the pharmacokinetics of PTCy used to prevent GVHD in pediatric patients. Pharmacokinetics, i.e., the absorption, distribution, metabolism, and excretion of drugs in the body, can vary with patient age. Owing to their rapidly changing physiology, pediatric patients may respond differently to drugs than adults. Studies have reported that the PKs of Cy administered after transplantation may differ between pediatric and adult patients. This could potentially lead to age-based differences in GVHD rates and overall survival based on age [16]. Therefore, it is important to consider the potential age-related differences in drug effects and safety when directly applying dosages established for adults to pediatric and adolescent patients.

Therefore, this study aimed to determine the appropriate dose and administration of high-dose PTCy following haploidentical HSCT in pediatric patients. We aimed to compare and analyze these results with previously reported PK data in adults. Additionally, we sought to confirm the correlation between PK outcomes and clinical complications.

## Methods

### Study population and study design

We conducted a prospective PK analysis of Cy in pediatric patients undergoing haploidentical HSCT, employing a myeloablative busulfan-based conditioning regimen with PTCy at Seoul National University Children’s Hospital (SNUCH). All enrolled patients were aged < 19 years and had high-risk malignancies or immunodeficiencies necessitating allogeneic HSCT.

Cyclophosphamide was administered intravenously for 2 h before and after HSCT. For PK analysis, 2 mL blood samples were collected in EDTA tubes at 2, 3, 6, and 24 h from the initiation of cyclophosphamide infusion on days − 3 and 3, and at 2, 3, and 6 h on days − 2 and 4. Fourteen samples were collected from each patient for PK analysis, and the total blood volume was intended to remain below 2.4 mL/kg. In one case, where the patient weighed 10.1 kg, 1.7 mL blood samples were collected.

This study was approved by the Institutional Review Board of Seoul National University Hospital (H-2103-193-1208 and H-2405-151-1539) and written informed consent was obtained from the legal guardians of all participating patients before any study procedures commenced.

### Transplantation protocol

All patients received a uniform myeloablative conditioning regimen, which included PK-targeted busulfan (administered intravenously once daily from days − 9 to -6), fludarabine (40 mg/m^2^ intravenously once daily from days − 8 to -4), and Cy (14.5 mg/kg intravenously once daily from days − 3 to -2). On day − 9, busulfan was administered at a dose of 120 mg/m^2^ for patients aged one year or older, and 80 mg/m^2^ for those under the age of one year. Subsequently, the targeted busulfan dose was adjusted based on the previous day’s therapeutic drug monitoring results and was administered from days − 8 to -6. The desired range for the busulfan area under the curve was set at 74 to 76 mg × h/L, in accordance with previous studies [7, 12].

For GVHD prophylaxis, PTCy (50 mg/kg intravenously once daily on days + 3 and + 4), tacrolimus (continuously intravenously from days + 5 to + 20 and orally twice a day from days + 21 to + 240), and mycophenolate mofetil (orally twice a day from days + 5 to + 35) were administered. Prophylactic measures for veno-occlusive disease (VOD), infection control, and regular screening for cytomegalovirus, Epstein-Barr virus, BK virus, and Aspergillus followed our institutional guidelines for hematopoietic stem cell transplantation [7, 17].

### Definition of engraftment, GVHD, and toxicities

Neutrophil engraftment was defined as the first of three consecutive days with an absolute neutrophil count of > 0.5 × 10^9^/L. Platelet engraftment was defined as the first day with a platelet count of > 20 × 10^9^/L, provided that no platelet transfusion had been received in the previous 7 days. For two patients who died of disease progression or treatment-related mortality before platelet engraftment, the day of platelet engraftment could not be determined.

Regimen-related toxicity was assessed and graded according to the National Cancer Institute Common Toxicity Criteria (version 5.0). Toxicity data were collected from the initiation of the conditioning regimen to 42 days after HSCT. The diagnosis and grading of acute and chronic GVHD were based on standard criteria [18, 19]. Veno-occlusive disease diagnostic criteria and severity were based on the European Society for Blood and Marrow Transplantation (EBMT) criteria [20].

### Determination of the plasma concentration

Plasma concentrations of Cy and its metabolite carboxycyclophosphamide (CCP) were analyzed using a high-performance liquid chromatography mass spectrometry system in the positive ionization mode, in accordance with the bioanalytical method validation guidance for industry from the U.S. Food and Drug Administration. Cy-d4 and CCP-d4 were used as the internal standards for Cy and CCP, respectively. The accuracy and precision of the quality check samples were within 15%. The quantitative ranges for cyclophosphamide and CCP were 100–10,000 and 10–1,000 ng/ml, respectively.

### Noncompartmental PK analysis

The PK parameters were estimated using non-compartmental methods by Phoenix WinNonlin^®^ software version 8.4 (Pharsight Co, Mountain View, CA, USA). The maximum concentration (C_max_), area under the concentration-time curve from dosing to the time of the last measured concentration (AUC_last_), area under the concentration-time curve within the dosing interval (AUC_tau_, tau = 24 h) calculated using the linear-up/log-down trapezoidal method, time to reach C_max_ (T_max_), half-life (t_1/2_), total clearance (CL), and volume of distribution (V_z_) were determined for each dosing day. Additionally, the metabolic ratio was calculated as the ratio of the AUC_last_ of cyclophosphamide to that of CCP.

### Population PK analysis

Population PK models of Cy and its metabolites HCY and CEPM were developed using NONMEM version 7.5 (Icon Development Solutions, Ellicott City, MD, USA). After reviewing the PK profile of Cy, the base models, including one-, 2-compartment model, were evaluated. The PK parameters of HCY and CEPM were adopted from a previously reported model in pediatric patients [21]. The model incorporated inter-individual variability and was developed using first-order conditional estimation with interaction (FOCE-I). The covariate analysis included age, weight, height, body surface area (BSA), sex, BUN, AST, ALT, total bilirubin, ferritin, eGFR, and busulfan exposure. The covariate was selected when its inclusion decreased the objective function value to 3.84 and met physiological plausibility. The model was evaluated using goodness-of-fit (GOF) plots and a visual predictive check (VPC). Using the final model, the PK profiles of HCY and CEPM were simulated and compared with the simulated PK profile of the adult population. When simulating pediatric patients, the demographic features of pediatric patients in SNUCH who underwent a myeloablative conditioning regimen, including busulfan, between 2009 and 2020 were utilized. The virtual adult population was assumed to be 60 kg and was simulated 1000 times for each dose. Simulated PTCy doses were adopted from previous studies [22–25].

### Statistical analysis

All data are presented as descriptive statistics. The PK parameters of patients with and without complications were compared using the Mann-Whitney U test. The PK parameters included the cumulative AUC_last_, cumulative AUC_tau_ and maximum C_max_ from day − 3 to day 4. All analyses were performed using R software version 4.3.1 (R Foundation for Statistical Computing, Vienna, Austria).

## Results

### Patients’ characteristics

Fifteen pediatric patients were enrolled in this study. The general characteristics of the patients are summarized in Table 1. Among the 15 patients, 13 had malignant diseases, including high-risk leukemia (*n* = 11), myelodysplastic syndrome (*n* = 1), and relapsed/refractory Ewing sarcoma (*n* = 1). The remaining 2 patients were diagnosed with chronic granulomatous disease accompanied with recurrent infections, which required allogeneic HSCT. As none of the patients had matched siblings or unrelated donors, haploidentical HSCT was performed using peripheral blood as the stem cell source. The median age at HSCT was 9.6 years (range, 1.6–16.8), and nine patients were female. Three patients had a history of allogeneic or autologous HSCT, and all patients exhibited normal renal and hepatic function according to their laboratory results. There were no unexpected adverse events during the Cy infusion period for any of the patients.

**Table 1:** Patients’ characteristics

Characteristics	*N* = 15
Age, year (range)	9.6 (1.6–16.8)
Sex, n	
Male	6 (40.0%)
Female	9 (60.0%)
Body weight, kg (range)	29.9 (10.1–73.0)
Height, cm (range)	132.0 (79.2-168.6)
Body surface area, m2 (range)	1.1 (0.5–1.8)
Diagnosis, n	
Acute myeloid leukemia	7 (46.7%)
Acute lymphoblastic leukemia	4 (26.7%)
Other malignancies	2 (13.3%)
Chronic granulomatous disease	2 (13.3%)
Donor, n	
Father	5 (33.3%)
Mother	4 (26.7%)
Sister	4 (26.7%)
Brother	2 (13.3%)
Previous transplantation, n	
Yes	3 (20.0%)
No	12 (80.0%)
Busulfan AUC, mg*h/L (range)	75.8 (73.4–79.1)
Pre-HSCT serum lab (range)	
BUN (mg/dL)	10.0 (6.0–14.0)
Creatinine (mg/dL)	0.44 (0.32–0.64)
Cystatin C (mg/L)	0.69 (0.42–0.89)
AST (IU/L)	25.0 (13.0–41.0)
ALT (IU/L)	22.0 (7.0–55.0)
Total bilirubin (mg/dL)	0.7 (0.4-1.0)
Ferritin (ng/mL)	1,414 (10 − 6,834)
Follow-up time, days (range)	416 (106–769)

ALT, alanine transaminase; AST, aspartate transaminase; AUC, area under the curve; BUN, blood urea nitrogen; n, number

### Engraftment, complications, GVHD, and survival

All patients achieved successful neutrophil engraftment at a median of 14 days post-HSCT (range: 10–21 days). Among the 13 evaluable patients, the median time to platelet engraftment was 40 days (range: 13–84 days).

Regarding post-HSCT complications, 11 (73.3%) patients were diagnosed with severe VOD. Although the incidence of VOD was unexpectedly high in our cohort, all patients successfully recovered after defibrotide administration, with a median treatment duration of 25 days (range, 11–39). The median onset of severe VOD diagnosis was 18 days post-HSCT (range, 12–24). According to the EBMT criteria [20], Patients were diagnosed with severe VOD based on elevated liver function tests exceeding five times the normal value (*n* = 6), refractory thrombocytopenia lasting more than 7 days (*n* = 3), or elevated total bilirubin levels (≥ 2 mg/dL, *n* = 2) (Supplementary Table 1) It was challenging to determine whether the criteria for severe VOD were solely attributable to VOD itself or were potentially influenced by concurrent infections or drug use. Nevertheless, we adhered to the same treatment regimen with defibrotide, and all patients successfully recovered from VOD.

Six patients (40.0%) experienced grade 3 or higher elevations in hepatic enzymes, and 3 patients (20.0%) had grade 2 or higher serum creatinine elevations. Additionally, three patients (20.0%) developed grade 3 hemorrhagic cystitis, which was attributed to BK virus infection. All post-HSCT complications were managed effectively and resolved.

In terms of GVHD, two patients had acute GVHD, graded 2 and 4, respectively, with skin involvement as the sole affected organ. The cumulative incidence of acute GVHD grade 2 or higher in our cohort was 13.3%. Four patients experienced mild (*n* = 2) or moderate (*n* = 2) chronic GVHD affecting organs such as the skin, oral mucosa, and lungs. However, no cases of extensive chronic GVHD were observed. The 1-year cumulative incidence of moderate-to-severe chronic GVHD in our cohort was 14.7%. During the follow-up period, three patients died: two due to disease progression and one due to sepsis (Table 2).

**Table 2:** Clinical outcomes

Variables	*n* = 15
Engraftment, yes	15 (100%)
Engraftment days	
Neutrophil	14 (10–21)
Platelet (*n* = 13)	40 (13–84)
Acute GVHD, yes	2 (13.3%)*
Chronic GVHD, yes	4 (26.7%)
Mild	2 (13.3%)
Moderate	2 (13.3%)
Severe	0 (0.0%)
Complications	
Severe VOD	11 (73.3%)
LFT elevation ≥ grade 3	6 (40.0%)
Total bilirubin ≥ grade 3	1 (6.7%)
Creatinine elevation ≥ grade 2	3 (20.0%)
Hemorrhagic cystitis ≥ grade 3	3 (20.0%)
Survival	
Alive	12 (80.0%)
Dead	3 (20.0%)

* Grade 2 (*n* = 1), Grade 4 (*n* = 1)

GVHD, graft-versus-host disease; LFT, liver function test; VOD, veno-occlusive disease;

### Noncompartmental PK analysis result

**Fig. 1:**
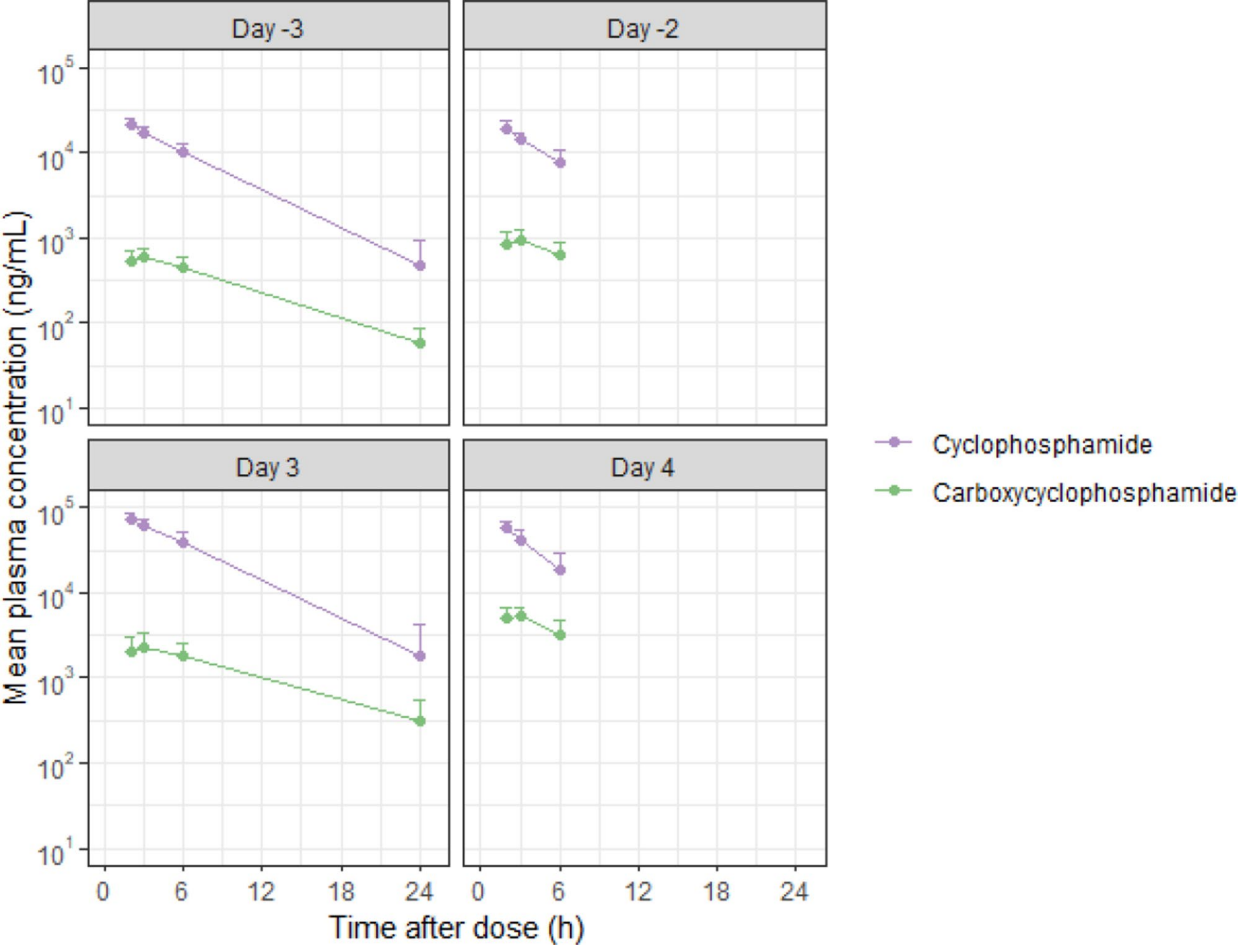
Mean plasma concentration-time profiles of cyclophosphamide and carboxycyclophosphamide in semi log scale. Bars represent the standard deviations

The plasma concentration of Cy peaked immediately after the end of infusion and was eliminated following a mono-exponential pattern. The PK parameters of individual patients were influenced by demographic factors, and CL and V_z_ tending to expand with increasing weight, height, and BSA (Supplementary Table 2). The PK parameters of Cy were similar between the first and third doses (days − 3 and 3) and between the second and fourth doses (days − 2 and 4). Cyclophosphamide CL increased from 3.5 L/h on day − 3 to 4.8 L/h on day − 2 before HSCT and doubled on day 4 compared to day 3 (Table 3). Plasma CCP concentrations reached C_max_ one hour after the end of infusion and were eliminated following a mono-exponential pattern. The CCP C_max_ values after the second dose were higher than those after the first dose, particularly after HSCT (days 3 and 4). There was a 30% increase in the metabolic ratio on day − 2 compared to day − 3, and it more than doubled on day 4 compared to day 3, with a value of 12.1% (Table 3; Fig. 1).

**Table 3:** Pharmacokinetic parameters of cyclophosphamide and carboxycyclophosphamide

	Parameter	Day − 3	Day − 2	Day 3	Day 4
		Cy 14.5 mg/kg/day		Cy 50 mg/kg/day	
Cy	C_max_ (mg/L)	20.93 (15.12–27.29)	19.23 (12.53–28.25)	72.34 (49.26–91.62)	57.75 (42.98–84.95)
	AUC_last_ (mg/L ∙ h)	131.7 (73.22-204.35)	66.05 (43.02-102.59)	486.13 (231.99-839.41)	189.89 (116.55-318.98)
	AUC_tau_ (mg/L ∙ h)	134.8 (75.49-205.76)	107.84 (59.2–177)	495.76 (232.1-851.52)	285.2 (143.15–731.3)
	T_max_ (h)	2.2 (2.05–3.12)	2.1 (1.95–2.25)	2.1 (2-2.22)	2.1 (2.03.2.3)
	t_1/2_ (h)	3.8 (2.6–6.3)	3.0 (1.8–5.1)	3.7 (2.4–6.7)	2.4 (1.6–5.8)
	V_z_ (L)	19.1 (7.6–36.7)	20.0 (8.2–38.7)	17.3 (7.1–29.9)	21.6 (7.6–40.9)
	CL (L/h)	3.5 (1.7–6.2)	4.8 (2.0-8.9)	3.3 (1.5–5.6)	6.6 (2.2–13.4)
CCP	C_max_ (mg/L)	0.61 (0.26–0.89)	0.94 (0.5–1.55)	2.33 (0.94–4.9)	5.29 (3.6-9)
	AUC_last_ (mg/L ∙ h)	6.01 (3.42–9.17)	3.96 (2.16–6.3)	24.59 (13.20-42.02)	22.03 (15.09–38.08)
	T_max_ (h)	3.1 (2.05–6.07)	3.0 (2.05–5.98)	3.5 (2.-5.98)	3.0 (2.03–5.92)
	t_1/2_† (h)	6.5 (4.3–13.8)	9.7 (3.1–20.3)	5.8 (4.1–8.2)	4.8 (3.2–7.1)
	Metabolic ratio (%)	4.8 (2.2–7.3)	6.3 (3.0-11.2)	5.4 (2.4–10.8)	12.1 (5.7–18.7)

Notes: Data are displayed as mean (range)

Abbreviations: Cy, cyclophosphamide; CCP, carboxycyclophosphamide

† t_1/2_ of CCP was estimated in 14, 3, 12, and 3 patients on day − 3, day − 2, day 3, and day 4, respectively

### Relationships between the post-HSCT complications and Cy PK parameters

**Fig. 2:**
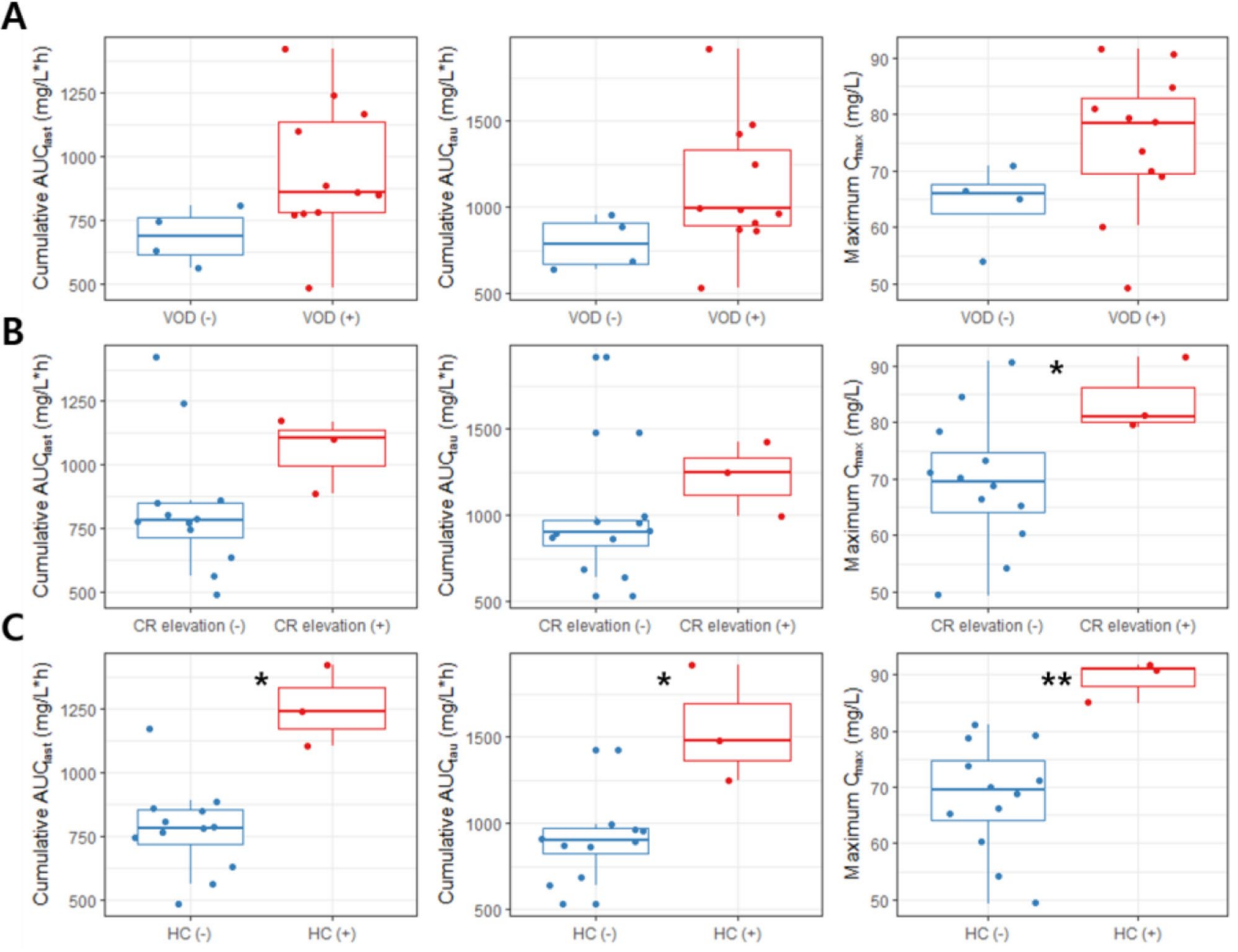
Comparison of the pharmacokinetic (PK) parameters of cyclophosphamide between patient groups with and without complications: (**A**) severe VOD (**B**) creatinine elevation ≥ grade 2 (**C**) hemorrhagic cystitis ≥ grade 3

**Fig. 3:**
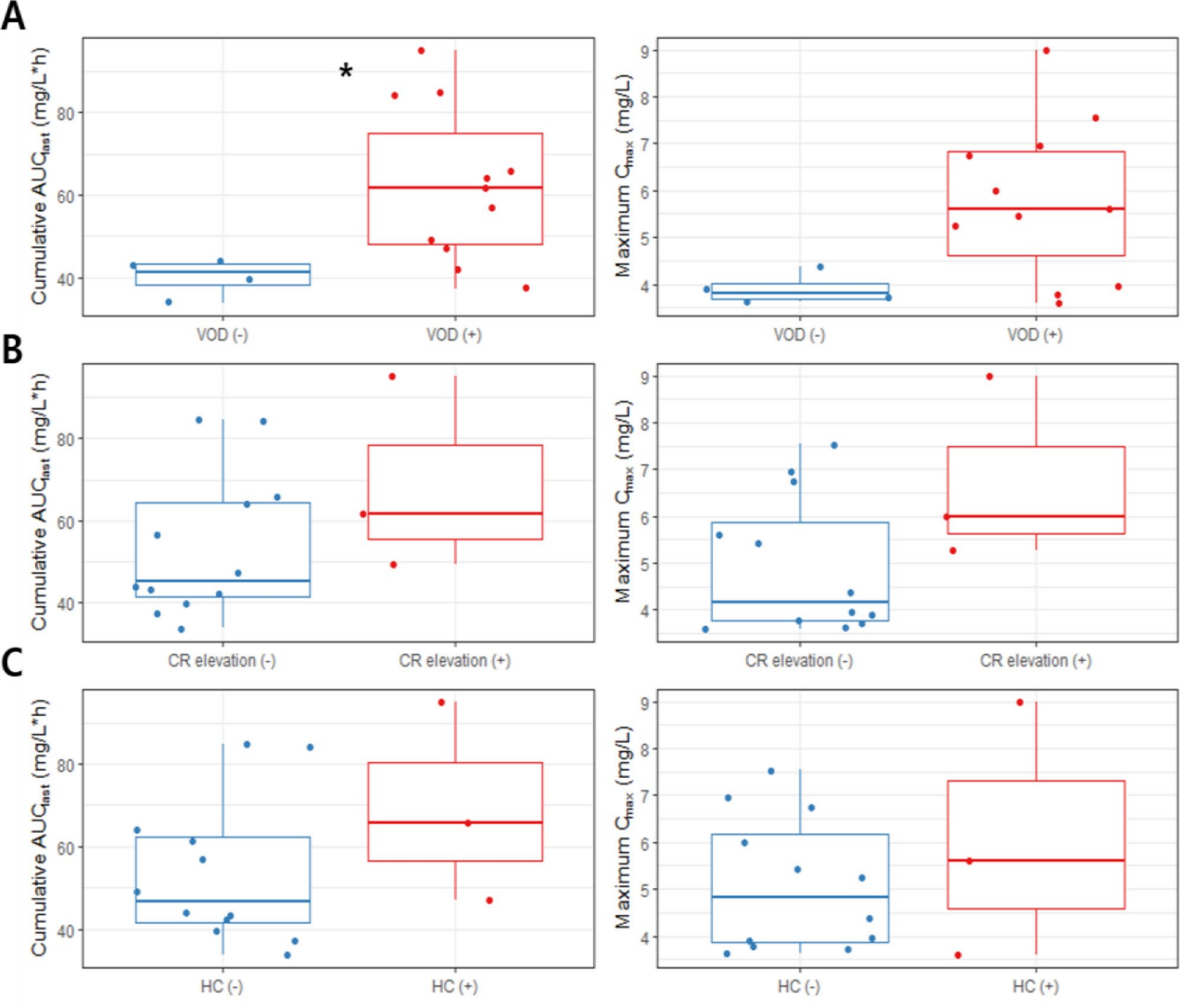
Comparison of the pharmacokinetic (PK) parameters of carboxycyclophosphamide between patient groups with and without complications: (**A**) severe VOD (**B**) creatinine elevation ≥ grade 2 (**C**) hemorrhagic cystitis ≥ grade 3

Patients with severe VOD, grade 2 or higher serum creatinine levels, and hemorrhagic cystitis had higher Cy concentrations. The cumulative AUC_last_, cumulative AUC_tau_, and maximum C_max_ during treatment periods in patients with the severe VOD were 941.66 mg/L ∙ h, 1108.05 mg/L ∙ h, and 75.3 mg/L, respectively and those were 687.04 mg/L ∙ h, 791.35 mg/L ∙ h, and 64.2 mg/L, respectively in patients without severe the VOD (Table 4; Fig. 2). The same pattern was observed for the pharmacokinetic parameters of CCP, and the cumulative AUC_last_ was significantly higher in patients with severe VOD than in those without severe VOD (Table 5, P-value < 0.05, Fig. 3). The maximum C_max_ of cyclophosphamide was significantly higher in patients with grade 2 or higher serum creatinine elevations than in patients without such complications (Table 5, P-value < 0.05). In addition, the cumulative AUC_last_, cumulative AUC_tau_, and maximum C_max_ were significantly higher in patients with grade 3 or higher hemorrhagic cystitis than in those without this complication (Table 5; all *P*-values < 0.01).

In contrast, there were no notable differences in the cumulative AUC_last_, cumulative AUC_tau_, and maximum C_max_ of Cy and CCP between the groups with or without acute GVHD and chronic GVHD. While not statistically significant, patients experiencing acute or chronic GVHD exhibited a tendency toward elevated cumulative AUC_last_ and cumulative AUC_tau_ levels of Cy.

**Table 4:** Comparison of cyclophosphamide Pharmacokinetic parameters between patient groups with and without complications

Complications PK parameters	Patients with complication	Patients without complication	*p*-value†
**Acute GVHD**	*n* = 2	*n* = 13	
Cumulative AUC_last_ (mg/L ∙ h)	1083.97 ± 480.15	841.42 ± 223.74	0.6857
Cumulative AUC_tau_ (mg/L ∙ h)	1403.39 ± 726.24	965.16 ± 280.73	0.4762
Maximum C_max_ (mg/L)	75.08 ± 13.82	71.92 ± 12.72	0.9333
**Chronic GVHD**	*n* = 4	*n* = 11	
Cumulative AUC_last_ (mg/L ∙ h)	951.07 ± 317.88	845.65 ± 244.43	0.7531
Cumulative AUC_tau_ (mg/L ∙ h)	1170.77 ± 498.46	970.08 ± 307	0.5714
Maximum C_max_ (mg/L)	72.24 ± 11.42	72.38 ± 13.26	0.8513
**Severe VOD**	*n* = 11	*n* = 4	
Cumulative AUC_last_ (mg/L ∙ h)	941.66 ± 265.95	687.04 ± 109.18	0.0557
Cumulative AUC_tau_ (mg/L ∙ h)	1108.05 ± 379.56	791.35 ± 153.99	0.104
Maximum C_max_ (mg/L)	75.3 ± 12.78	64.2 ± 7.17	0.104
**LFT elevation ≥ grade 3**	*n* = 6	*n* = 9	
Cumulative AUC_last_ (mg/L ∙ h)	870.46 ± 306.85	875.97 ± 240.4	0.8639
Cumulative AUC_tau_ (mg/L ∙ h)	1034.33 ± 464.49	1016.44 ± 301.27	1
Maximum C_max_ (mg/L)	72.6 ± 12.5	72.17 ± 13.07	0.7756
**Total bilirubin ≥ grade 3**	*n* = 1	*n* = 14	
Cumulative AUC_last_ (mg/L ∙ h)	770.77	881.12 ± 266.29	0.6667
Cumulative AUC_tau_ (mg/L ∙ h)	872.51	1034.39 ± 370.19	0.6667
Maximum C_max_ (mg/L)	68.94	72.58 ± 12.82	0.8
**CR elevation ≥ grade 2**	*n* = 3	*n* = 12	
Cumulative AUC_last_ (mg/L ∙ h)	1054.26 ± 147.17	828.64 ± 264.18	0.1011
Cumulative AUC_tau_ (mg/L ∙ h)	1219.52 ± 218.27	974.61 ± 377.41	0.1363
Maximum C_max_ (mg/L)	83.95 ± 6.7	69.44 ± 11.89	0.0484^*^
**HC ≥ grade 3**	*n* = 3	*n* = 12	
Cumulative AUC_last_ (mg/L ∙ h)	1256.23 ± 160.33	778.15 ± 174.05	0.0088^*^
Cumulative AUC_tau_ (mg/L ∙ h)	1545.81 ± 341.42	893.04 ± 223.65	0.0088^*^
Maximum C_max_ (mg/L)	89.12 ± 3.72	68.15 ± 9.84	0.0044^**^

Notes Data are presented as mean ± standard deviation. Maximum C_max_ was defined as the C_max_ throughout day − 3 to day 4

Abbreviations: PK, pharmacokinetics; GVHD, graft-versus-host disease; VOD, venous occlusive disease; CR, creatinine; HC, hemorrhagic cystitis

† Mann-Whitney test

* *P*-value < 0.05

Boxes represent the interquartile range (25th to 75th percentile, IQR), horizontal lines represent the median, vertical lines represent the minimum to maximum between the range of 1.5 times IQR, and closed circles represent the observed values. Asterisks indicate statistical significance (*P*-value < 0.05: *, *P*-value < 0.005: **).

Abbreviations: VOD, venous occlusive disease; CR, creatinine; HC, hemorrhagic cystitis.

**Table 5:** Comparison of carboxycyclophosphamide PK parameters between patient groups with and without complications

Complications PK parameters	Patients with complication	Patients without complication	*p*-value†
**Acute GVHD**	*n* = 2	*n* = 13	
Cumulative AUC_last_ (mg/L ∙ h)	49.87 ± 22.63	57.62 ± 19.23	0.6857
Maximum C_max_ (mg/L)	4.61 ± 1.41	5.4 ± 1.74	0.5714
**Chronic GVHD**	*n* = 4	*n* = 11	
Cumulative AUC_last_ (mg/L ∙ h)	55.44 ± 24.2	57 ± 18.14	0.7531
Maximum C_max_ (mg/L)	4.98 ± 1.59	5.41 ± 1.77	0.7531
**Severe VOD**	*n* = 11	*n* = 4	
Cumulative AUC_last_ (mg/L ∙ h)	62.55 ± 18.77	40.17 ± 4.58	0.0264^*^
Maximum C_max_ (mg/L)	5.8 ± 1.68	3.9 ± 0.33	0.0557
**LFT elevation ≥ grade 3**	*n* = 6	*n* = 9	
Cumulative AUC_last_ (mg/L ∙ h)	63.84 ± 17.76	51.75 ± 19.21	0.181
Maximum C_max_ (mg/L)	5.79 ± 1.28	4.96 ± 1.89	0.181
**Total bilirubin ≥ grade 3**	*n* = 1	*n* = 14	
Cumulative AUC_last_ (mg/L ∙ h)	64.1	56.05 ± 19.6	0.6667
Maximum C_max_ (mg/L)	6.73	5.19 ± 1.69	0.5333
**CR elevation ≥ grade 2**	*n* = 3	*n* = 12	
Cumulative AUC_last_ (mg/L ∙ h)	68.61 ± 23.69	53.58 ± 17.55	0.1802
Maximum C_max_ (mg/L)	6.75 ± 1.98	4.93 ± 1.46	0.233
**HC ≥ grade 3**	*n* = 3	*n* = 12	
Cumulative AUC_last_ (mg/L ∙ h)	69.32 ± 24.17	53.4 ± 17.26	0.1802
Maximum C_max_ (mg/L)	6.07 ± 2.93	5.1 ± 1.42	0.8396

Notes Data are presented as mean ± standard deviation. Maximum C_max_ was defined as the C_max_ throughout day − 3 to day 4

Abbreviations: PK, pharmacokinetics; GVHD, graft-versus-host disease; VOD, venous occlusive disease; CR, creatinine; HC, hemorrhagic cystitis

† Mann-Whitney test

* *P*-value < 0.05

Boxes represent the interquartile range (25th to 75th percentile, IQR), horizontal lines represent the median, vertical lines represent the minimum to maximum between the range of 1.5 times IQR, and closed circles represent the observed values. The number of asterisks indicates statistical significance (*P*-value < 0.05).

Abbreviations: VOD, venous occlusive disease; CR, creatinine; HC, hemorrhagic cystitis.

### Population PK analysis result

**Fig. 4:**
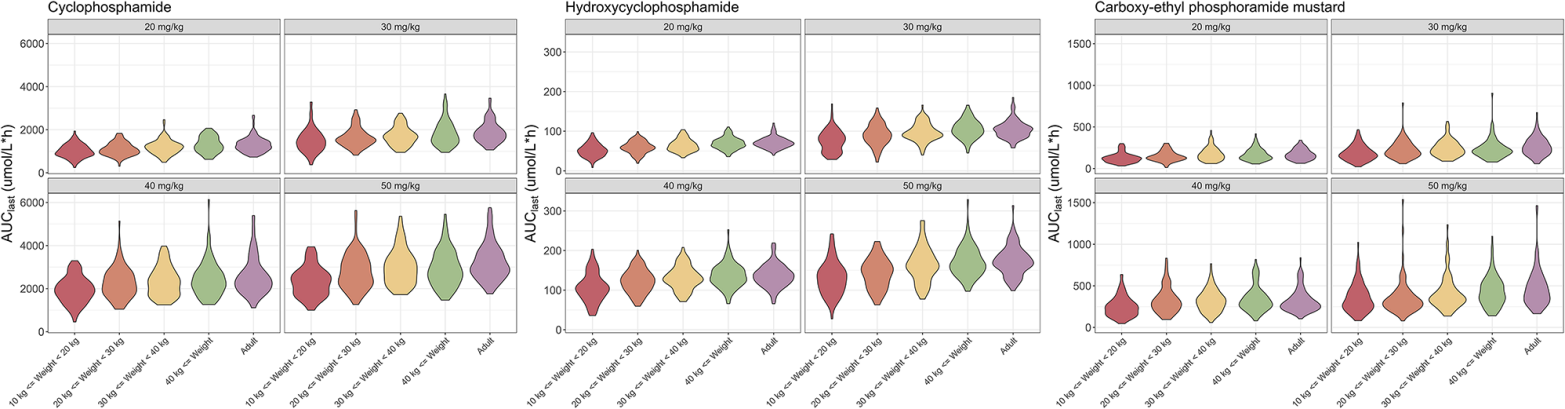
Simulated area under the curve (AUC) of cyclophosphamide, hydroxycyclophosphamide, and carboxy-ethyl phosphoramide mustard in pediatric and adult patients

The PK profile of Cy was well-explained by a 1-compartment with inducible (CL_IND_) and non-inducible (CL_NON_) clearance models (Supplementary Fig. 1). Weight was considered a significant covariate for V_z_ and the CL_IND_ of Cy. Table 6 presents the estimates and relative standard errors of the PK parameters for Cy, HCY, and CEPM. The GOF plots and VPC showed that the final model adequately described the PK profiles of Cy (Supplementary Fig. 2). As weight was regarded as a significant covariate in the PK model, the distribution profile of body weight in pediatric patients in a real-world data setting was applied during the simulation (Supplementary Fig. 3). In the high-weight group, exposure to CY, HCY, and CEPM increased (Fig. 4). The mean AUC of HCY and CEPM were similar between pediatric patients weighing > 30 kg and the adult population (Fig. 4, Supplementary Table 3).

**Table 6:** Parameter Estimation of the final Pharmacokinetic model of cyclophosphamide and its metabolite

Parameter	Final model		
	Estimate	RSE (%)	Shrinkage
Population parameters			
Volume of distribution for cyclophosphamide (L/kg)	0.636	4	
Clearance			
CL_IND_ (L/h/kg)	0.0346	10	
CL_NON_ (L/h)	0.715	20	
Enzyme degradation rate (K_ENZ_, h^− 1^)	0.0572	12	
Volume of distribution for HCY (L/kg)^*^	Fixed at 0.013		
HCY elimination rate (K_HCY_, h^− 1^)^*^	Fixed at 147		
CEPM formation rate from HCY (K34, h^− 1^)^*^	Fixed at 2.23		
Volume of distribution for CEPM (L/kg)^*^	Fixed at 0.013		
CEPM elimination rate (K_CEPM_, h^− 1^)^*^	Fixed at 0.948		
Cyclophosphamide concentration of half maximal induction (EC50, umol/L)^*^	Fixed at 0.6		
Maximum induction (E_MAX_)^*^	Fixed at 5		
**Inter-individual variability**			
Clearance			
CL_IND_ (%)	39.8	22	7
CL_NON_ (%)	82.4	26	25
Volume of distribution for cyclophosphamide (%)	12	17	8
**Residual Error**†			
Proportional residual error	0.0853	28	
Additive residual error	5.17	29	

Abbreviations: RSE, residual Standard Error calculated as (standard error)/(estimate) × 100; Vz, volume of distribution; CL_IND_, inducible clearance of cyclophosphamide; CL_NON_, non-inducible clearance of cyclophosphamide; HCY, hydroxycyclophosphamide; CEPM, carboxy-ethyl phosphoramide mustard

† Residual error of cyclophosphamide

* Pharmacokinetic parameters adopted from a previously reported model

## Discussion

Our study provides comprehensive PK profiles of Cy in pediatric patients who underwent haploidentical HSCT using a myeloablative conditioning regimen and pharmacokinetic-targeted busulfan. Exploring the relationship between PK profiles of Cy in pediatric patients and clinical outcomes ensures optimal dose selection, as increasing doses can enhance the efficacy of a drug, but may also be accompanied by toxicity.

The PK profile of Cy before and after HSCT in pediatric patients showed a monoexponential pattern in the terminal phase, which has also been described in previous articles (Fig. 1) [21, 26, 27]. Increase in CL and metabolic ratio of Cy on day − 2 and day 4 implied the induction in its metabolizing process (Table 3). The auto-induction of Cy has been proposed in many other studies, and some have incorporated it into their population PK models [14, 26, 28]. The maximal induction potential (E_max_) and half the concentration of Cy for maximal induction (EC50) has been proposed by Qiu et al. as 5 and 0.6 umol/L [21]. Ever since, many researchers have adopted these values and used them in Cy enzyme turnover models [14, 27, 29]. We also fixed the E_max_ and EC50 with the same value and found that the model adequately described the PK profile of Cy (Table 6). Weight was selected as a significant covariate in the population PK model of Cy. We collected the weight distribution data of pediatric patients who underwent a myeloablative conditioning regimen at SNUCH and used them for PK simulation. We expect that the use of real-world data will significantly contribute to the accurate prediction of the actual PK profiles of Cy and its metabolites.

In previous studies, HCY and CEPM have been the Cy metabolites most commonly explored. However, HCY and CEPM are highly unstable and have been reported to show considerable variability across previous studies [30–32]. In this study, the concentration of CCP was measured as an alternative because of the difficulty in setting up procedures for measuring HCY and CEPM. There was a significantly higher exposure to CCP in patients with severe VOD (Table 5), as in previous studies, and a higher exposure to HCY and CEPM was observed in patients with VOD [33, 34]. CCP is generated during the metabolic pathway in which CY is converted to HCY, which subsequently transforms into aldophosphamide and eventually into CEPM or CCP. This supports CCP’s role as a metabolic marker of Cy and the biomarker for VOD prediction. Furthermore, as far as we know, this is the first study introducing the PK profile of CCP. CCP reached T_max_ one hour the cessation of infusion, which was explained by a one-compartment PK model.

In our study, neither the AUC nor the C_max_ of Cy was associated with the incidence of acute and chronic GVHD (Table 4). Plasma Cy concentrations were higher in patients with post-HSCT complications, such as grade 2 or higher serum creatinine elevations or grade 3 or higher hemorrhagic cystitis (Table 4). This implies that the dose of PTCy used in this study (50 mg/kg) may have been excessive. The limited number of subjects might have been insufficient to verify the results, considering the large inter-individual variability in Cy exposure, and there is a need for future studies with a larger sample size.

Adjustment of the PTCy dosage is an actively explored area of research. Efforts have been made to decrease the dose of Cy, which has resulted in manageable GVHD rates and positive outcomes [22, 35, 36]. Additionally, employing half the standard PTCy dose (25 mg/kg on days 3 and 5) demonstrated favorable outcomes in pediatric patients with Fanconi anemia [37]. Depending on the patient characteristics, reducing the PTCy dose could be a viable option to mitigate HSCT toxicities, particularly in cases involving a myeloablative conditioning regimen. However, studies assessing the efficacy and safety of a myeloablative conditioning regimen with reduced doses of PTCy in pediatric patients are scarce. Our study showed that children weighing > 30 kg exhibited PK similar to those in adults (Fig. 4 and Supplementary Table 3). This suggests that it might be feasible to reduce the PTCy dose in this pediatric population based on adult study results to mitigate toxicity. For children weighing less than 30 kg, our study observed a tendency for a reduced AUC of Cy and its metabolites compared with adults. However, there were no significant differences in VOD or GVHD based on body weight, although the small sample size limited the interpretation. Given that the PK-pharmacodynamic relationship in pediatric patients could differ [38, 39], more careful consideration is needed before suggesting that children weighing less than 30 kg require more Cy to achieve comparable drug exposure to that of adults. Additional prospective studies are needed to evaluate the appropriate PTCy dose for pediatric patients with diverse body weights.

At our institution, we implemented PK monitoring-targeted busulfan-based conditioning in pediatric patients undergoing haploidentical HSCT with PTCy. Although optimizing busulfan dosage has led to successful engraftment and positive outcomes in both malignant and non-malignant cases [7, 12] the use of high-dose double alkylating agents carries inherent risks, particularly the elevated risk of VOD, which remains a significant concern. According to the literature, the incidence of pediatric VOD ranges from 15 to 30%, depending on factors such as age, underlying disease, and conditioning regimens [40, 41]. Among these cases, severe VOD occurs in 30–40%, with mortality rates of up to 80% [42, 43]. However, in this study, the incidence of severe VOD was unexpectedly high (73.3%), whereas the mortality rate associated with VOD was 0%. One possible explanation for this discrepancy is the increased sensitivity of the EBMT VOD criteria we applied in our cohort [44]. Although these patients were diagnosed with severe VOD based on elevated liver function tests or refractory thrombocytopenia, these conditions could also potentially be associated with concurrent infections or drug use at the time. Furthermore, the small cohort size limits the generalizability of the VOD incidence observed at our institution, which has been reported as 14–20% in previous studies [7, 12]. Notably, only one patient required renal replacement therapy, and all patients successfully recovered with defibrotide administration. However, VOD should be carefully monitored in patients receiving high-dose double alkylating agents such as busulfan and PTCy. Considering the correlation between Cy PK parameters and HSCT complications, excluding GVHD, the PK-based dose adjustments for PTCy warrant further investigation in this patient population.

In summary, our study provides insights into the PK profiles of Cy and CCP in pediatric patients undergoing haploidentical HSCT with PTCy. The small sample size certainly limits the generalizability of these results; however, the intensive PK assessments per patient may help overcome this limitation. The intricate interplay between PK parameters and post-HSCT complications suggests the need for tailored adjustments in PTCy dosage, particularly in pediatric patients subjected to myeloablative conditioning regimens.

## Electronic supplementary material

Below is the link to the electronic supplementary material.

Supplementary Material 1: Supplementary Fig. 1. Illustration of the population pharmacokinetic model of cyclophosphamide and its metabolite. Cyclophosphamide is metabolized to HCY, which is then metabolized to CEPM. An enzyme compartment was used to explain the auto-induction of cyclophosphamide metabolism. The inducible clearance (CL_IND_) depends on the concentration of cyclophosphamide (C_CY_). Supplementary Fig. 2. Goodness-of-fit plots and visual predictive checks for the final pharmacokinetic model of cyclophosphamide. (A) Goodness-of-fit plots: Open circles indicate observations; solid black lines are identity lines. (B) Visual predictive checks: Circles represent observed concentrations; solid lines represent the 5th (blue), median (red), and 95th (blue) concentration percentiles; blue and red areas indicate the 90% confidence interval of the simulated concentrations of each percentile. Supplementary Fig. 3. Weight distribution of pediatric patients undergoing myeloablative conditioning regimen in Seoul National University Children’s Hospital from 2009 to 2020.

Supplementary Material 2

## Data Availability

No datasets were generated or analysed during the current study.

## Abbreviations

PTCy: Post-transplantation cyclophosphamide
GCHD: Graft-versus-host disease
PK: Pharmacokinetic
HSCT: Haploidentical hematopoietic stem cell transplantation
CEPM: Carboxy-ethyl phosphoramide mustard
HCY: Hydroxycyclophosphamide
SNUCH: Seoul National University Children’s Hospital
IIV: Inter-individual variability
BSA: Body surface area
GOF: Goodness-of-fit
VPC: Visual predictive check
