# Correction: Pharmacokinetics of post-transplant cyclophosphamide and its associations with clinical outcomes in pediatric haploidentical hematopoietic stem cell transplantation

**DOI:** 10.1186/s40364-025-00781-3

**Published:** 2025-04-27

**Authors:** Kyung Taek Hong, Sungyeun Bae, Yoon Sunwoo, Juyeon Lee, Hyun Jin Park, Bo Kyung Kim, Jung Yoon Choi, Joo-Youn Cho, Kyung-Sang Yu, Jaeseong Oh, Hyoung Jin Kang

**Affiliations:** 1 Department of Pediatrics, Seoul National University College of Medicine, Seoul National University Children’s Hospital, Seoul, Republic of Korea; 2 Seoul National University Cancer Research Institute, Seoul, Republic of Korea; 3 Department of Clinical Pharmacology and Therapeutics, Seoul National University College of Medicine and Hospital, Seoul, Republic of Korea; 4 Department of Biomedical Sciences, Seoul National University College of Medicine, Seoul, Republic of Korea; 5 Department of Pharmacology, Jeju National University College of Medicine, Jeju, 63241 Republic of Korea; 6 Wide River Institute of Immunology, Hongcheon, Republic of Korea

**Biomarker Research (2025) 13:48**

**https://doi.org/10.1186/s40364-025-00749-3**

The original article has been amended to restore last author, Hyoung-Jin Kang (kanghj@snu.ac.kr) as a co-Corresponding Author.

