# Supplementary material for: Pharmacokinetics of post-transplant cyclophosphamide and its associations with clinical outcomes in pediatric haploidentical hematopoietic stem cell transplantation
Source: Biomark Res. 2025 Mar 24;13:48. doi: 10.1186/s40364-025-00749-3 (PMC11934747; doi:10.1186/s40364-025-00749-3)
Supplement: Supplementary file 1 — Supplementary Material 1: Supplementary Fig. 1. Illustration of the population pharmacokinetic model of cyclophosphamide and its metabolite. Cyclophosphamide is metabolized to HCY, which is then metabolized to CEPM. An enzyme compartment was used to explain the auto-induction of cyclophosphamide metabolism. The inducible clearance (CLIND) depends on the concentration of cyclophosphamide (CCY). Supplementary Fig. 2. Goodness-of-fit plots and visual predictive checks for the final pharmacokinetic model of cyclophosphamide. (A) Goodness-of-fit plots: Open circles indicate observations; solid black lines are identity lines. (B) Visual predictive checks: Circles represent observed concentrations; solid lines represent the 5th (blue), median (red), and 95th (blue) concentration percentiles; blue and red areas indicate the 90% confidence interval of the simulated concentrations of each percentile. Supplementary Fig. 3. Weight distribution of pediatric patients undergoing myeloablative conditioning regimen in Seoul National University Children’s Hospital from 2009 to 2020. [file 40364_2025_749_MOESM1_ESM.pptx]

## Slide 1
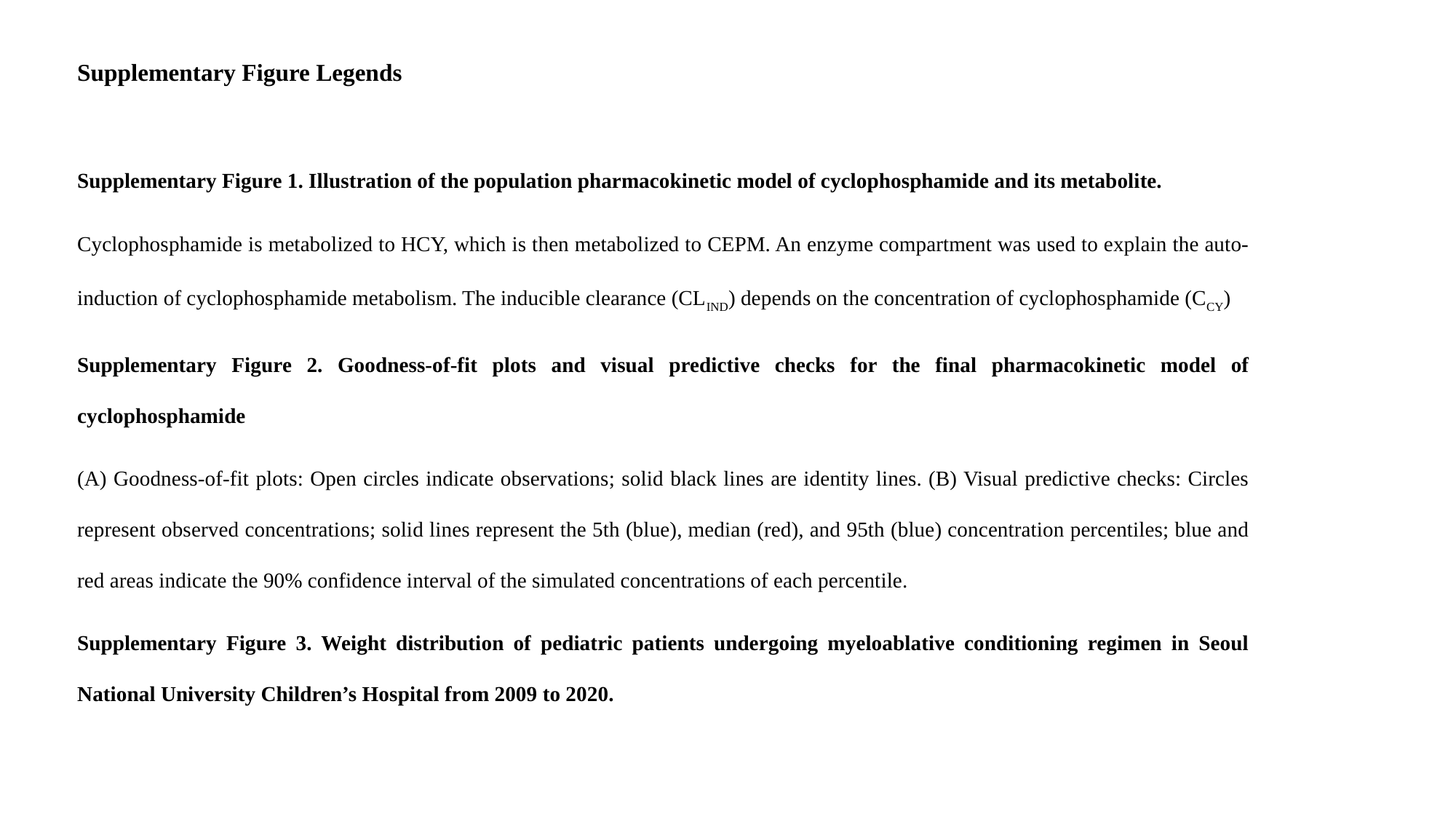

Supplementary Figure Legends
Supplementary Figure 1. Illustration of the population pharmacokinetic model of cyclophosphamide and its metabolite.
Cyclophosphamide is metabolized to HCY, which is then metabolized to CEPM. An enzyme compartment was used to explain the auto-induction of cyclophosphamide metabolism. The inducible clearance (CLIND) depends on the concentration of cyclophosphamide (CCY)
Supplementary Figure 2. Goodness-of-fit plots and visual predictive checks for the final pharmacokinetic model of cyclophosphamide
(A) Goodness-of-fit plots: Open circles indicate observations; solid black lines are identity lines. (B) Visual predictive checks: Circles represent observed concentrations; solid lines represent the 5th (blue), median (red), and 95th (blue) concentration percentiles; blue and red areas indicate the 90% confidence interval of the simulated concentrations of each percentile.
Supplementary Figure 3. Weight distribution of pediatric patients undergoing myeloablative conditioning regimen in Seoul National University Children’s Hospital from 2009 to 2020.

## Slide 2
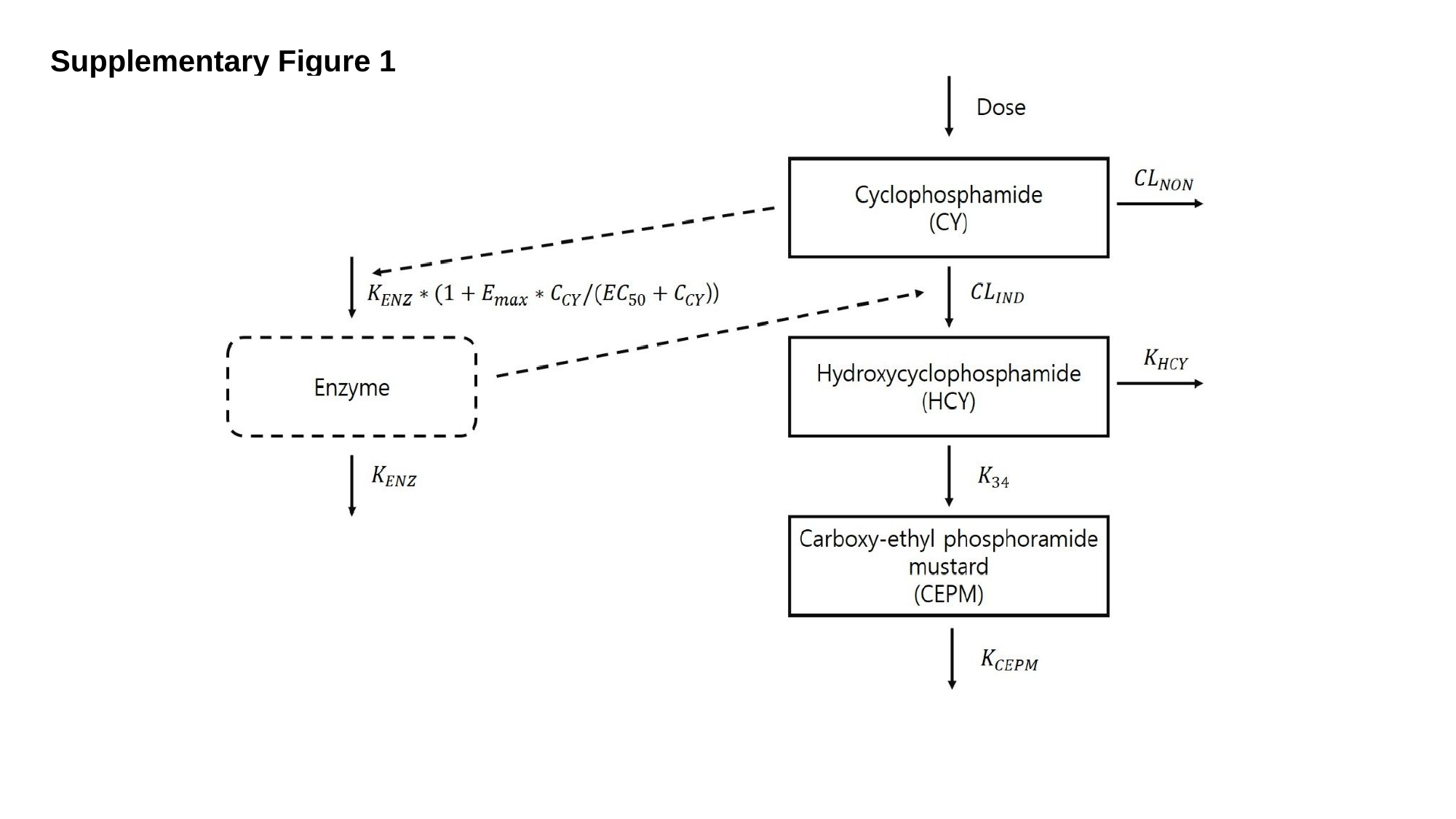

Supplementary Figure 1

## Slide 3
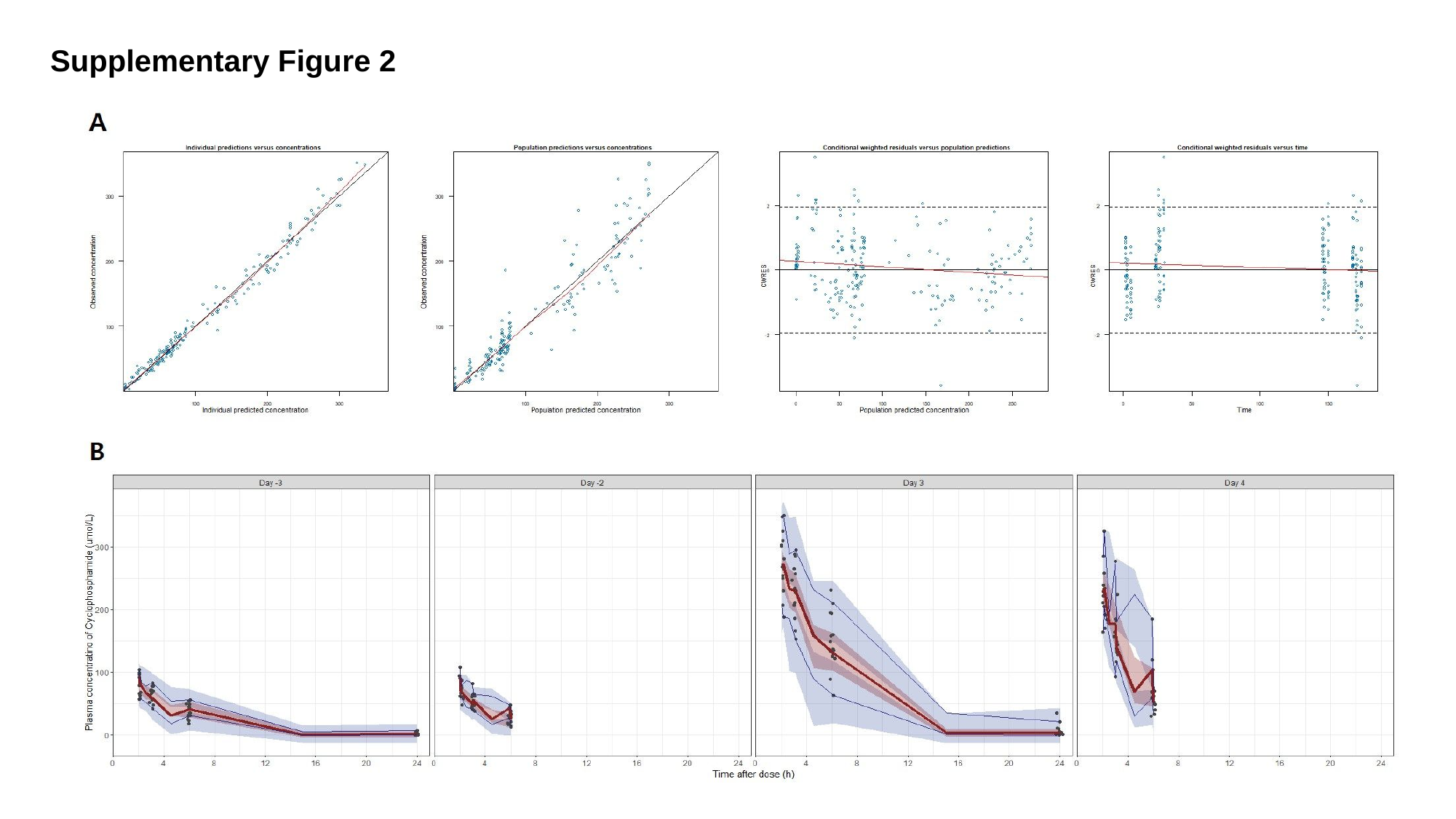

Supplementary Figure 2

## Slide 4
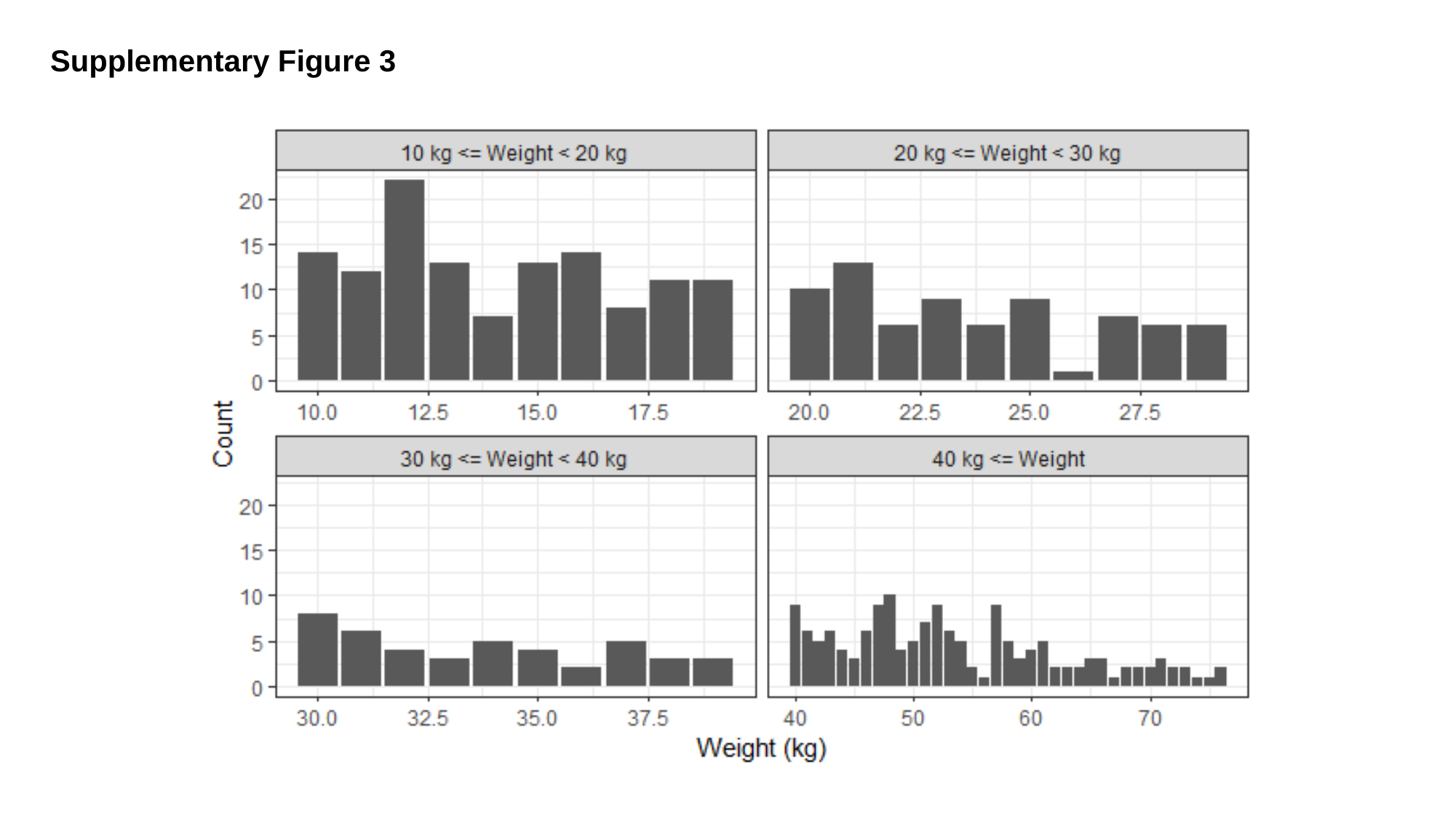

Supplementary Figure 3
