# Supplementary material for: Pharmacokinetics of post-transplant cyclophosphamide and its associations with clinical outcomes in pediatric haploidentical hematopoietic stem cell transplantation
Source: Biomark Res. 2025 Mar 24;13:48. doi: 10.1186/s40364-025-00749-3 (PMC11934747; doi:10.1186/s40364-025-00749-3)
Supplement: Supplementary file 2 — Supplementary Material 2 [file 40364_2025_749_MOESM2_ESM.docx]

**Supplementary Table 1. Detailed information of patients treated for VOD**

| Patient No. | Diagnosis | Criteria met for the diagnosis of VOD | Criteria met for severe VOD diagnosis | The onset of VOD from HSCT | The duration of defibrotide treatment | VOD outcome |
| --- | --- | --- | --- | --- | --- | --- |
| 2 | AML | Refractory thrombocytopenia, Body weight gain, Hematpomegaly, Ascites | Refractory thrombocytopenia >7days | day 24 | 16 days | resolved |
| 4 | AML | Refractory thrombocytopenia, Hematpomegaly, Bilirubin | Total Bilirubin 2.6 mg/dL | day 13 | 39 days | resolved |
| 5 | AML | Refractory thrombocytopenia, Hematpomegaly, Ascites | Refractory thrombocytopenia >7days | day 12 | 26 days | resolved |
| 6 | ALL | Body weight gain, Hematpomegaly, Ascites | AST 279 IU/L, ALT 108 IU/L | day 18 | 29 days | resolved |
| 7 | AML | Body weight gain, Ascites | AST 300 IU/L, ALT 306 IU/L | day 21 | 11 days | resolved |
| 8 | ALL | Refractory thrombocytopenia, Body weight gain, Hematpomegaly, Ascites | AST 280 IU/L, ALT 164 IU/L | day 20 | 25 days | resolved |
| 9 | ALL | Body weight gain, Hematpomegaly, Ascites | AST 372 IU/L, ALT 330 IU/L | day 21 | 32 days | RRT required, resolved |
| 10 | ALL | Refractory thrombocytopenia, Body weight gain, Hematpomegaly, Ascites | AST 1000 IU/L, ALT 610 IU/L | day 14 | 18 days | resolved |
| 11 | Ewing sarcoma | Hematpomegaly, Ascites | AST 337 IU/L, ALT 564 IU/L | day 16 | 13 days | resolved |
| 13 | AML | Hematpomegaly, Ascites, Bilirubin | Total Bilirubin 2.1 mg/dL | day 17 | 15 days | resolved |
| 14 | MDS | Refractory thrombocytopenia, Body weight gain, Ascites | Refractory thrombocytopenia >7days | day 20 | 25 days | resolved |

ALL, Acute lymphoblastic leukemia; ALT, alanine transaminase; AML, Acute myeloid leukemia; AST, aspartate transaminase; HSCT, Hematopoietic stem cell transplantation; MDS, Myelodysplastic syndrome; RRT, Renal replacement therapy

**Supplementary Table 2. Mean pharmacokinetic parameters throughout the study of cyclophosphamide according to the demographic characteristics of individual patients**

| No. | Age  (year) | Sex | Body weight  (kg) | Height  (cm) | Body surface area  (m^2^) | CL  (L/h) | V_z_  (L) | t_1/2_  (h) |
| --- | --- | --- | --- | --- | --- | --- | --- | --- |
| 1 | 6.3 | F | 20.1 | 115.2 | 0.80 | 4.31 | 14.07 | 2.48 |
| 2 | 13.8 | M | 73 | 160 | 1.80 | 8.50 | 36.03 | 3.19 |
| 3 | 10.5 | F | 29.85 | 139.1 | 1.07 | 4.13 | 18.94 | 3.51 |
| 4 | 8.2 | F | 27.1 | 125.2 | 0.97 | 5.12 | 16.39 | 2.43 |
| 5 | 6.5 | M | 20.85 | 116.7 | 0.82 | 3.36 | 14.42 | 3.30 |
| 6 | 14.6 | F | 47.6 | 157.4 | 1.44 | 6.38 | 29.29 | 3.37 |
| 7 | 9.6 | M | 39.75 | 132 | 1.21 | 6.56 | 22.36 | 2.47 |
| 8 | 5.1 | M | 20.65 | 105.8 | 0.78 | 5.17 | 15.11 | 2.10 |
| 9 | 16.8 | F | 45.35 | 156.6 | 1.40 | 6.23 | 24.42 | 2.95 |
| 10 | 4.9 | F | 17.65 | 104.7 | 0.72 | 2.80 | 10.35 | 2.69 |
| 11 | 11.3 | F | 31.7 | 141.7 | 1.12 | 2.39 | 16.74 | 5.11 |
| 12 | 2.7 | M | 12.3 | 86.3 | 0.54 | 1.97 | 8.77 | 3.22 |
| 13 | 14.3 | F | 49.3 | 149.6 | 1.43 | 4.35 | 23.28 | 3.91 |
| 14 | 13.1 | F | 54.05 | 168.6 | 1.59 | 5.05 | 34.62 | 5.09 |
| 15 | 1.4 | M | 10.1 | 79.2 | 0.47 | 2.21 | 7.69 | 2.46 |

CL, clearance; Vz, volume of distribution; t_1/2_, half-life.

**Supplementary Table 3. Simulated area under the curve of cyclophosphamide, hydroxycyclophosphamide (HCY), and carboxy-ethyl phosphoramide mustard (CEPM) in pediatric and adult patients.**

|  | 20 mg/kg | 30 mg/kg | 40 mg/kg | 50 mg/kg |
| --- | --- | --- | --- | --- |
| Cyclophosphamide |  |  |  |  |
| 10 kg≤Weight<20 kg | 989.49 | 1443.96 | 1924.70 | 2304.64 |
| 20 kg≤Weight<30 kg | 1063.83 | 1553.45 | 2208.00 | 2690.93 |
| 30 kg≤Weight<40 kg | 1188.84 | 1682.40 | 2278.20 | 2919.89 |
| 40 kg≤Weight | 1259.58 | 1726.73 | 2303.65 | 2855.90 |
| Adult | 1265.76 | 1791.05 | 2392.95 | 3066.83 |
| HCY |  |  |  |  |
| 10 kg≤Weight<20 kg | 50.71 | 76.90 | 104.29 | 128.53 |
| 20 kg≤Weight<30 kg | 59.26 | 90.22 | 122.81 | 147.04 |
| 30 kg≤Weight<40 kg | 63.37 | 94014 | 128.33 | 162.49 |
| 40 kg≤Weight | 67.34 | 104.96 | 135.07 | 166.39 |
| Adult | 69.79 | 103.97 | 135018 | 170.71 |
| CEPM |  |  |  |  |
| 10 kg≤Weight<20 kg | 122.33 | 173.53 | 239.46 | 310.69 |
| 20 kg≤Weight<30 kg | 135.30 | 204.60 | 301.19 | 308.04 |
| 30 kg≤Weight<40 kg | 160.29 | 228.50 | 317.80 | 354.48 |
| 40 kg≤Weight | 150.46 | 214.43 | 312.14 | 401.91 |
| Adult | 158.43 | 256.26 | 286.78 | 427.92 |

Notes: Data are presented as the median value of AUC_last_ (umol/L ∙ h)
